# Optimized Approaches for the Induction of Putative Canine Induced Pluripotent Stem Cells from Old Fibroblasts Using Synthetic RNAs

**DOI:** 10.3390/ani10101848

**Published:** 2020-10-11

**Authors:** Mirae Kim, Seon-Ung Hwang, Junchul David Yoon, Yeon Woo Jeong, Eunhye Kim, Sang-Hwan Hyun

**Affiliations:** 1 Veterinary Medical Center and College of Veterinary Medicine, Laboratory of Veterinary Embryology and Biotechnology (VETEMBIO), Chungbuk National University, Cheongju 28644, Korea; kmr9309@naver.com (M.K.); ghkdsun@hanmail.net (S.-U.H.); jdyoon86@hanmail.net (J.D.Y.); 2 Institute of Stem Cell & Regenerative Medicine (ISCRM), Chungbuk National University, Cheongju 28644, Korea; 3 Abu Dhabi Biotech Research Foundation, 64 Kyungin-ro, Guro-gu, Seoul, Korea; doctorj1@adbrf.org

**Keywords:** canine, reprogramming, iPSCs, VEE RNA, integration-free

## Abstract

**Simple Summary:**

A non-integrating and self-replicating *Venezuelan equine encephalitis* RNA replicon system can potentially make a great contribution to the generation of clinically applicable canine induced pluripotent stem cells. Our study shows a new method to utilize the synthetic RNA-based approach for canine somatic cell reprogramming regarding transfection and reprogramming efficiency.

**Abstract:**

Canine induced pluripotent stem cells (ciPSCs) can provide great potential for regenerative veterinary medicine. Several reports have described the generation of canine somatic cell-derived iPSCs; however, none have described the canine somatic cell reprogramming using a non-integrating and self-replicating RNA transfection method. The purpose of this study was to investigate the optimal strategy using this approach and characterize the transition stage of ciPSCs. In this study, fibroblasts obtained from a 13-year-old dog were reprogrammed using a non-integrating *Venezuelan equine encephalitis* (VEE) RNA virus replicon, which has four reprogramming factors (collectively referred to as *T7-VEE-OKS-iG* and comprised of *hOct4*, *hKlf4*, *hSox2*, and *hGlis1*) and co-transfected with the *T7-VEE-OKS-iG* RNA and *B18R* mRNA for 4 h. One day after the final transfection, the cells were selected with puromycin (0.5 µg/mL) until day 10. After about 25 days, putative ciPSC colonies were identified showing TRA-1-60 expression and alkaline phosphatase activity. To determine the optimal culture conditions, the basic fibroblast growth factor in the culture medium was replaced with a modified medium supplemented with murine leukemia inhibitory factor (mLIF) and two kinase inhibitors (2i), PD0325901(MEK1/2 inhibitor) and CHIR99021 (GSK3β inhibitor). The derived colonies showed resemblance to naïve iPSCs in their morphology (dome-shaped) and are dependent on mLIF and 2i condition to maintain an undifferentiated phenotype. The expression of endogenous pluripotency markers such as *Oct4, Nanog*, and *Rex1* transcripts were confirmed, suggesting that induced ciPSCs were in the late intermediate stage of reprogramming. In conclusion, the non-integrating and self-replicating VEE RNA replicon system can potentially make a great contribution to the generation of clinically applicable ciPSCs, and the findings of this study suggest a new method to utilize the VEE RNA approach for canine somatic cell reprogramming.

## 1. Introduction

The derivation of induced pluripotent stem cells (iPSCs) using four reprogramming factors known as Yamanaka’s factors (*Oct4*, *Sox2*, *Klf4*, and *c-Myc*) opens a new avenue for patient-specific regenerative medicine therapies [1,2,3]. As public understanding of animal welfare and health issues increases, this technology can be also applied in veterinary medicine. Dogs, the most representative companion animals, are very useful model for the development of new animal therapeutics such as gene and stem cell-based therapies [4,5,6]. Canine iPSCs (ciPSCs) show great potential not only for veterinary regenerative medicine but also for translational medicine as a disease model [7,8]. While many studies have used rodent models for human disease, they are poor representations of the human system [9], but dogs are a more applicable model for a number of reasons. Most practically, the lifespan of dogs is longer than that of rodents, which allows long-term studies [10]. Dogs also have a similar size and similar genomic, anatomical, and physiological characteristics to those of humans [11]. It is important to note that the average age of companion dogs has increased in recent years, and that dogs share some disease-related genes with humans. In particular, common canine diseases share similarities with human diseases, such as cancers, autoimmune diseases, and diabetes [12,13,14,15].

To date, several reports have described the generation of ciPSCs using retroviral or lentiviral transduction using Yamanaka’s factors [7,8,16]. Although viral reprogramming is very useful and conventionally performed, numerous studies have shown that it can induce genomic integration and increase the tumorigenic potential [17,18]. Due to these potential problems associated with integration, viral reprogramming is not suitable for clinical applications. For instance, one reprogramming factor *c-Myc* can increase the reprogramming efficiency, but it is a proto-oncogene that can induce tumor formation [19,20,21]. Consequently, it has long been debated whether *c-Myc* can be safely used as a reprogramming factor. Instead, the glis-family zinc finger 1 (*Glis1*) gene, which encodes a Krüppel-like protein, was found to have the ability to directly reprogram somatic cells into iPSCs more fully than the *c-Myc* gene [22]. Unlike *c-Myc*, the *Glis1* gene poses no increased risk for tumor formation and efficiently suppresses the proliferation of colonies that have not been fully reprogrammed. Hence, using the *Glis1* gene for reprogramming may be a good alternative instead of the *c-Myc* gene.

Recently, many kinds of integration-free methods for generating iPSCs without *c-Myc* have been developed [18,23]. For example, the *Sendai* virus, episome (Epi), and synthetic mRNA-transfection-based methods can avoid potential integration problems. Especially, there have recently been two reports of canine somatic cell reprogramming into iPSCs using the replication-defective and persistent *Sendai* virus (SeVdp) vector [24,25]. However, it is not easy to use SeVdp vectors for the regulation of reprogramming gene expressions, and they are too expensive to use frequently. In previous studies, a polycistronic and synthetic self-replicating RNA system was developed to generate human iPSCs using the RNA replicon from of *Venezuelan equine encephalitis* (VEE) virus [26,27]. The VEE replicon is a single-stranded positive sense RNA that contains a 5′ cap and poly (A) tail, similar to cellular mRNAs. The VEE replicon has no potential for problems associated with genomic DNA integration, because it does not use a DNA intermediate [28,29,30]. It is also easier and more productive to synthesize RNA than to produce *Sendai* virus particles with higher biosafety in the laboratory.

Although several reports have described the generation of ciPSCs [7,16,24,25,31,32], little is known about the characteristics of the early stages in the process of canine cell reprogramming from somatic cells to pluripotent stem cells. Unlike mouse and human cells, a few reports have characterized the final pluripotent stage of ciPSCs [10,31]. In mice, a genetic study was conducted to analyze progression from the transition stage to naïve embryonic stem cells [33], which reaffirmed the intermediate phase of pluripotency and helped to understand the molecular dynamics of the transition state. Likewise, several studies have analyzed the generation of authentic human pluripotent stem cells from pre-pluripotent stem cells [34,35]. Data from these studies have shown that it is necessary to characterize transition-stage cell lines before applying iPSCs in clinical research. Furthermore, there remain several major limitations for the use of the generated ciPSCs as a reliable cell source. First, canine somatic cell reprogramming protocols were not well established and consistent with each other, resulting in an insufficient reproducibility [16]. Second, it has often been reported that they are difficult to maintain in long-term culture [8,36,37]. Particularly, there has been a continuous problem in the long-term culture of ciPSCs derived from the fibroblasts of a 13-year-old dog because the senescence easily occurs at passage 7 or more [38]. Third, the number of established ciPSC colonies is too low to be suitable for applications for the development of directed iPSC differentiation for broader clinical use (heart, neuron, muscle, etc.) [24,37].

To overcome these limitations, we have tried to reprogram canine adult fibroblasts (CAFs) into a pluripotent state by using an RNA transfection-based method, which is more reproducible and efficient for reprogramming [39]. Here, we first tried to canine somatic cell reprogramming using a synthetic and self-replicating RNA-based approach. We transfected in CAFs using an RNA strategy involving a non-infectious, self-replicating, and integration-free VEE virus that expresses four reprogramming open reading frames (ORFs) (*OKS-iG*; *hOct4, hKlf4, hSox2*, and *hGlis1*). CAFs were also co-transfected with *B18R* mRNA, which acts as an inhibitor of type-1 interferons to reduce the strong immune responses induced by VEE RNA [40,41]. Furthermore, we investigated the optimal conditions for the transfection and selection steps, and characterized the transition stage of putative ciPSCs.

## 2. Materials and Methods

### 2.1. Ethics Statement

This study was carried out in strict accordance with the recommendations in the Guide for the Care and Use of Laboratory Animals of the National Veterinary and Quarantine Service. The protocol was approved by the Committee on the Ethics of Animal Experiments of the Chungbuk National University (permit number: CBNUA-1415-20-02). All sacrifice was performed under isoflurane anesthesia, and all efforts were made to minimize suffering.

### 2.2. Chemicals

All the reagents were purchased from Sigma–Aldrich Chemical Company (St. Louis, MO, USA), unless stated otherwise.

### 2.3. Cell Culture

Canine skin tissue was isolated from a 13-year-old female Jindo dog. Primary CAFs were obtained from the Abu Dhabi Biotech Research Foundation (Seoul, Korea). The CAFs were cultured at 37 °C in Dulbecco’s Modified Eagle’s Medium (DMEM, high glucose) containing 10% fetal bovine serum (FBS), 1 × MEM non-essential amino acids, 1 × GlutaMAX, 0.1 mM β-mercaptoethanol, and 1 × antibiotic–antimycotic (all from Gibco, Carlsbad, CA, USA) in air containing 5% CO_2_.

### 2.4. Plasmid Construction and RNA Synthesis

To generate the *T7-VEE-mCherry* construct, *T7-VEE-OKS-iG* (#58974; Addgene, Cambridge, MA, USA) was linearized by NdeI/NotI digestion and modified by removing the *OKS-iG* sequence [42]. This vector was renamed as *T7-VEE*. *pSicoR-Ef1a-mCh-Puro* (#31845; Addgene) was digested with the same restriction enzymes, and then the mCherry-Puro sequence was introduced to the *T7-VEE* vector. The *pTNT-B18R* vector (#58978; Addgene) was amplified by PCR, and the amplicon was purified by gel extraction. These linearized DNA templates were used for in vitro transcription. The synthesis of *T7-VEE-mCherry* and *pTNT-B18R* RNA was performed with the RiboMAX Large Scale RNA Production System-T7 Kit (Promega, Madison, WI, USA). The ScriptCap m7G Capping System (Epicentre, Madison, WI, USA) was used for 5′ capping, which confers mRNA stability and efficient translation. After the 5′ mRNA- capping reaction, a poly (A) tail was added using poly (A) polymerase (Epicentre). These individual RNAs were purified by ammonium acetate and isopropanol precipitation, resuspended in the RNase-free water, and stored at −80 °C. A PCR was performed to confirm that both mRNAs were properly synthesized.

### 2.5. Determination of Optimal Puromycin Concentration

To determine the optimal puromycin concentration for selecting *VEE-OKS-iG* transfected cells, a puromycin resistance test was first performed on CAFs. CAFs were plated at a density of 5 × 10^4^ cells/mL in a 6-well dish and cultured in DMEM containing 0.25, 0.5, 0.8, 1, 1.5, and 2 µg/mL of puromycin (Puromycin dihydrochloride, P8833) for 7 days. The cell death of CAFs was observed by culturing with various concentration of puromycin for up to 7 days.

### 2.6. Measurement of Transfection Efficiencies by Flow Cytometry

Initially, CAFs were seeded in a 6-well plate at a density of 1 × 10^4^ cells/mL 24 h before transfection (Day 0). On day 1, the cells were transfected with *T7-VEE-mCherry* mRNA using Lipofectamine MessengerMAX (Thermo Fisher Scientific, Waltham, MA, USA) or the RiboJuice™ transfection reagent (EMD Millipore, Burlington, MA, USA). Both transfection reagents were used at 4 μL each and the RNA template was used at 1 μg each. The expression of *T7-VEE-mCherry* was measured by flow cytometry on day 2. CAFs were then washed once with DPBS (LB 001-02, Welgene, Gyeongsangbuk-do, Korea) and incubated with 0.25% trypsin–EDTA (Gibco) for 2 min in a 37 °C incubator. After dissociation and trypsin inactivation, these cells were resuspended with DPBS containing 1% FBS. The transfection efficiency was measured by a flow cytometry analysis using the SH800S cell sorter (Sony Imaging Products & Solutions, Tokyo, Japan) and analyzed with the cell sorter software version 1.8.3 (Sony Imaging Products and Solutions).

### 2.7. Preparation of Feeder Cells

Mouse embryonic fibroblasts (MEFs) were isolated from embryonic day 13.5 fetuses of the Institute of Cancer Research (ICR) strain. To inactivate the MEFs, they were treated with 10 μg/mL of mitomycin C (Roche, Basel, Switzerland) at passage 3 for 2 to 2.5 h before being used as feeder layers. The inactivated MEFs were plated at a density of 2.5 × 10^5^ cells/mL in 6-well dishes coated with 0.1% gelatin (EMD Millipore).

### 2.8. Preparation of Mouse Embryonic Fibroblast Conditioned Medium (MEF-CM)

To prepare the MEF-CM, inactivated MEFs were seeded at a concentration of 4 × 10^5^ cells/mL in 6-well dishes coated with 0.1% gelatin (EMD Millipore). The inactivated MEFs were cultured at 37 °C in DMEM containing 10% FBS, 1 × GlutaMAX, and 1 × antibiotic-antimycotic (all from Gibco) in air containing 5% CO_2_. The MEF-CM was collected on the next day and filtrated through a 0.2 μm syringe filter (Corning Incorporated, Corning, NY, USA).

### 2.9. Generation and Culture of the Putative ciPSCs

Reprogramming was performed using the Simplicon™ Reprogramming Kit (EMD Millipore). On day 0, the CAFs were seeded at a density of 5 × 10^4^ cells/mL in a 6-well plate coated with 0.1% gelatin (EMD Millipore). To minimize strong immune responses, CAFs were pre-treated with 200 ng/mL of human recombinant B18R protein (EMD Millipore) for 20 min before transfection. Transfection was performed on day 1 (1 × Tfx), on days 1 and 2 (2 × Tfx), or on days 1 to 4 (4 × Tfx). The cells were transfected with both *VEE-OKS-iG* RNA (EMD Millipore) and *B18R* RNA (EMD Millipore) using the RiboJuice™ mRNA Transfection Kit (EMD Millipore). After 4 h, the transfection medium was changed to stage 1 medium, consisting of Advanced-DMEM (Gibco) with 10% FBS (Gibco), 1 × GlutaMAX (Gibco), and 200 ng/mL of human recombinant B18R protein (EMD Millipore). The transfected cells were grown in stage 1 medium and selected with puromycin (0.5 µg/mL) until day 10. At day 10 post-transfection, the transfected cells were passaged onto inactivated MEFs using Accutase (Biowest, Riverside, MO, USA) in a 6-well plate (1 × 10^5^ cells/mL in each well). Stage 1 medium was used through day 10 and then it was replaced with stage 2 medium, comprised of MEF CM containing 10 ng/mL of basic fibroblast growth factor (bFGF; Bio-Bud, Gyunggido, Korea), 1 × human iPS reprogramming boost supplement II (TGF-β RI kinase inhibitor IV, sodium butyrate, and PS48) from the Simplicon™ Reprogramming Kit (EMD Millipore), and 200 ng/mL of B18R protein (EMD Millipore). The stage 2 medium was changed every other day. B18R protein (EMD Millipore) was added until small iPSC colonies appeared. Putative ciPSC colonies were mechanically isolated on days 22 to 30 and replated on inactivated MEFs in stage 3 medium, which consisted of DMEM/F12 medium, 20% KnockOut Serum Replacement medium (KSR), 1 × GlutaMAX, 1 × MEM NEAA, 0.1 mM of β-mercaptoethanol, and 1 × antibiotic–antimycotic (all from Gibco). Putative ciPSC colonies were cultured in stage 3 medium containing 10 ng/mL of bFGF (Bio-Bud) or 1000 units/mL of murine leukemia inhibitory factor (mLIF; ESGRO, Millipore, Billerica, MA, USA), plus 0.5 μM of MEK1/2 inhibitor (PD0325901; Stemgent, Cambridge, MA, USA) and 3 μM of GSK3β inhibitor (CHIR99021; Stemgent).

### 2.10. Primary iPSC Colony Staining with Alkaline Phosphatase (AP) and TRA-1-60

ciPSCs were washed with PBS three times after removing the iPSC culture medium from each 6-well plate, after which they were fixed in PBS containing 4% paraformaldehyde for 5 min at room temperature. Fixed cells were washed three times in PBS and stained with 5-bromo-4-chloro-3-indolyl phosphate/nitroblue tetrazolium (BCIP/NBT; Roche, Basel, Switzerland) solution for 30 min at room temperature in the dark. AP-positive cells were stained a dark purple color and visualized under a light microscope. For the TRA-1-60 staining, ciPSC colonies were washed with PBS three times, and colonies were stained using an Alexa Fluor 488-conjugated TRA-1-60 antibody (1:50) (Life Technologies, Carlsbad, CA, USA). After 1 h, the staining solution was removed and the cells were gently washed three times with FluoroBrite™ DMEM (Life Technologies). Images were acquired using a fluorescent microscope.

### 2.11. Gene Expression Analysis of Putative ciPSCs by Reverse Transcription PCR (RT-PCR)

The expression levels of *RPL13A* (a reference gene), *VEE-hOct4*, *VEE-hKlf4*, *VEE-hSox2*, *VEE-hGlis1*, *Rex1, Oct4*, and *Nanog* mRNA in ciPSCs were analyzed by RT-PCR. All the cDNA samples of ciPSC-like colonies were stored at −80 °C until PCR analysis. Total RNA was extracted from putative ciPSCs using the Trizol reagent (Invitrogen, Carlsbad, CA, USA). Complementary DNA (cDNA) was synthesized using Moloney murine leukemia virus (MMLV) reverse transcriptase (Invitrogen) and random primers (Invitrogen). All the procedures were performed in accordance with the manufacturer’s instructions. RT-PCR analysis was conducted using cDNA from putative ciPSCs. cDNA was amplified in a 20 μL PCR mixture consisting of 10 pmol of forward and reverse primers, 2 units of Taq polymerase, 2 µL of 10x PCR buffer, 5 pmol of dNTP mixtures (all from iNtRON Biotechnology, SungNam, Korea), and template DNA. The oligonucleotide primer sequences are presented in Table S1. PCR amplification was performed for 30 cycles of denaturation at 95 °C for 30 s, annealing at 57 °C for 30 s, and extension at 72 °C for 90 s. The reaction products were analyzed on a 1.25% agarose gel pre-stained with RedSafe™ Nucleic Acid Staining Solution (iNtRON Biotechnology).

### 2.12. Statistical Analysis

The statistical analyses were performed using SPSS 17.0 (SPSS, Inc., Chicago, IL, USA). The percentage data were compared using a one-way ANOVA followed by Tukey’s multiple range tests. All the results are expressed as the mean ± standard error of mean (SEM). *p* < 0.05 were considered to be statistically significant.

## 3. Results

### 3.1. Determination of Optimal Puromycin Concentration

**Figure 1:**
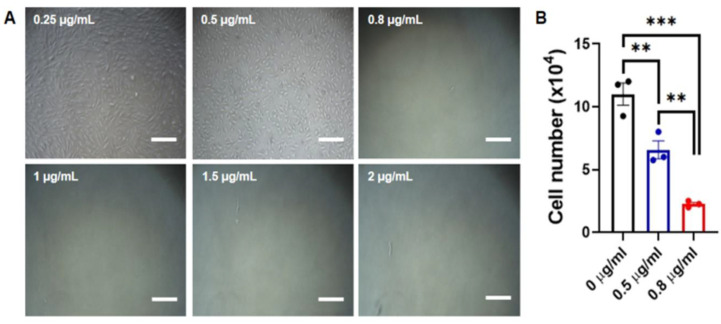
Determination of the optimal puromycin concentration for selecting transfected cells. (**A**) Representative images of various puromycin concentration-treated fibroblasts from a 13-year-old dog. Scale bars = 200 μm. (**B**) The number of cells counted on 4 days after the puromycin treatment. The value represents mean ± the standard error of the mean (SEM). Data were analyzed by one-way analysis of variance (ANOVA). Asterisks indicate statistical significance (** *p* < 0.01, *** *p* < 0.005).

Since the synthetic VEE RNA used in the experiment has a puromycin resistance gene, puromycin selection was performed to isolate the transfected cell line. Prior to transfecting synthetic RNAs, it is important to determine the optimal puromycin concentration for selecting transfected cells. For this, CAFs were plated at a density of 5 × 10^4^ cells/mL in a 6-well plate, after which they were cultured with various puromycin concentrations on day 1. After the addition of puromycin, varying levels of cell death were observed for 7 days. Surviving cells were not observed when exposed to puromycin concentrations higher than 0.5 μg/mL from day 4 (Figure 1A). The surviving cell number of the 0.8 μg/mL puromycin-treated group is significantly lower than that of the 0.5 μg/mL puromycin-treated group (Figure 1B). Therefore, it was determined that the treatment with 0.5 μg/mL of puromycin for 7 days enabled the most efficient selection of transfected CAFs. 

### 3.2. Synthesis of RNA by In Vitro Transcription (IVT) and Measurement of Transfection Efficiencies

**Figure 2:**
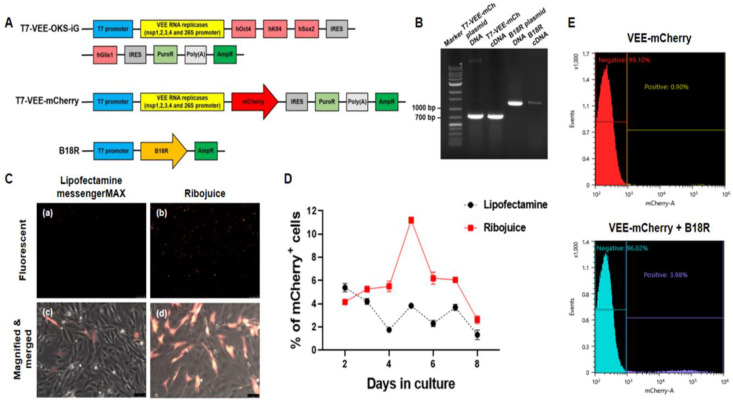
Construction of synthetic mRNAs and the measurement of transfection efficiencies. (**A**) Maps of *T7-VEE-OKS-iG*, *T7-VEE-mCherry,* and *B18R* vectors. *Venezuelan equine encephalitis* (VEE) RNA replicases are represented as bright yellow boxes. (**B**) Confirmation of plasmid DNA (control), and polymerase chain reaction (PCR) products (complementary DNA; cDNA) from RNA templates by reverse transcription (RT)-PCR analysis. (**C**) Representative images of *T7-VEE-mCherry* RNA-transfected canine fibroblasts comparing Lipofectamine messengerMAX and Ribojuice tranfection reagents. Scale bars = 250 μm for (a, b) and 75 μm for (c, d). (**D**) Percentage of *mCherry*^+^ transfected cells using the Lipofectamine MessengerMAX or RiboJuice transfection reagent. The value represents mean ± SEM. (**E**) Representative flow cytometry histogram showing the mCherry fluorescence of the *mCherry*^+^ transfected cell population. Transfected with VEE RNA alone (upper); co-transfected with the T7-VEE-mCherry RNA replicon and B18R (bottom). All the experiments were replicated at least three times.

To perform the mRNA-based reprogramming, the first step for the reprograming of CAFs is the production of synthetic RNA molecules encoding the reprogramming factors. The synthetic modified mRNAs containing each gene were produced as shown in Figure 2A. Synthetic *T7-VEE-mCherry* mRNA and *B18R* mRNA were synthesized by in vitro transcription (IVT). A non-infectious and self-replicating VEE RNA replicon was modified by removing the *OKS-iG* sequence. The VEE plasmid was linearized by NdeI/NotI digestion, and the fluorescent mCherry gene was introduced 3′ of the replicon ORF. The *B18R* plasmid was amplified by PCR, and the amplicon was purified by gel extraction. These linearized plasmids served as templates for RNA synthesis. RNA transcription was performed for 4 h at 37 °C with the RiboMAX Large Scale RNA Production System-T7 Kit. A 5′ capping enzyme and poly (A) polymerase were used to promote efficient translation. After the purification of the PCR product and the IVT, the cDNAs correctly synthesized from these RNAs were analyzed using agarose gel electrophoresis to determine the specific length and purity. The detected bands showed the expected lengths of the plasmid DNA (control) and PCR products (cDNA) from the RNA templates (Figure 2B). The sizes of the vectors used in the experiment are as follows: *T7-VEE-OKS-iG* is 16.86 kb, *T7-VEE-mCherry* is 11.477 kb, and *B18R* is 3.907 kb.

To determine which transfection reagent was more efficient, CAFs were transfected with *T7-VEE-mCherry* RNA on day 1, using the Lipofectamine MessengerMAX or the RiboJuice™ transfection reagent (Figure 2C). The results showed that the RiboJuice™ transfection reagent showed a higher percentage of mCherry^+^ cells than that of the Lipofectamine MessengerMAX-treated group (Figure 2D), which suggests that the use of the RiboJuice™ transfection reagent is a more optimized approach for the generation of ciPSCs. Additionally, CAFs were transfected with *T7-VEE-mCherry* RNA alone or co-transfected with *B18R* RNA using the RiboJuice™ transfection reagent. Because of the strong immune response induced by VEE RNA, few mCherry-positive cells were observed after transfection with *T7-VEE-mCherry* RNA alone. However, those cells co-transfected with the *T7-VEE-mCherry* RNA replicon and *B18R* RNA have shown more mCherry expression (3.98%) than the cells transfected with VEE RNA alone (0.9%) (Figure 2E). These results suggested that *B18R* is required for the more efficient transfection of VEE RNA replicons.

### 3.3. Generation of Putative ciPSCs Using Fibroblasts from an Aged Dog

**Figure 3:**
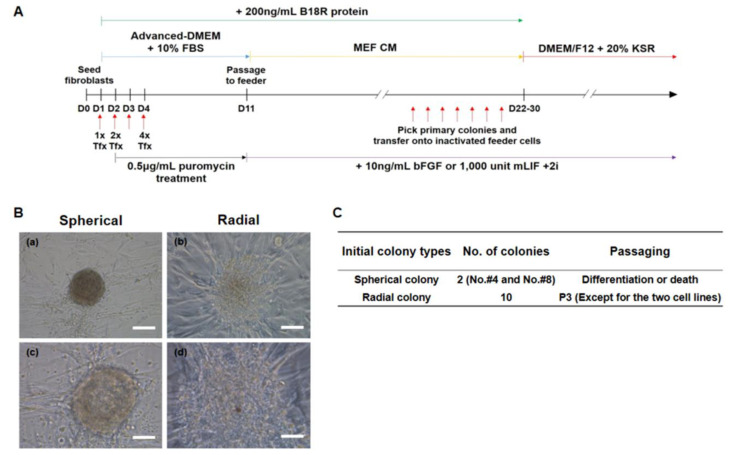
Two distinct types of initial canine induced pluripotent stem cell (ciPSC) colonies. (**A**) Timeline of synthetic mRNA-mediated reprogramming using a method of co-transfecting *T7-VEE-OKS-iG* and *B18R* mRNAs. (**B**) Representative images of the initial ciPSC colonies after introducing four reprogramming factors by VEE RNA on day 25. (a) and (c) Typical images of a spherical colony. (b) and (d) Typical images of a radial colony. Scale bars = 200 μm for (a, b); 50 μm for (c, d). (**C**) Information of the initial ciPSCs classified based on the colony morphology.

The synthetic mRNA-based reprogramming required the daily transfection of the cells to maintain a constant level of reprogramming factor expression. In this study, transfection was performed with the *T7-VEE-OKS-iG* replicon and *B18R* RNA on day 1 (1 × Tfx), days 1 and 2 (2 × Tfx), or days 1 to 4 (4 × Tfx) (Figure 3A). One day after the final transfection, the cells were selected with puromycin (0.5 µg/mL) until day 10. Then, 200 ng/mL of *B18R* protein was added to minimize the immune response until iPSC colonies appeared. To find the optimal reprogramming conditions, we have attempted to reprogram using a variety of culture media such as MEF-CM, Advanced-DMEM, or DMEM/F12. Among these conditions, the most effective culture condition was to use MEF-CM until the first colony appeared (for about 19 days) after VEE-OKS-iG mRNA transfection (data not shown). Twelve putative ciPSC colonies were derived from the CAFs and isolated on days 22 to 30. These initial colonies were of two distinct types, which are a spherical type and a radial type (Figure 3B). Two colonies showed a spherical-type morphology and 10 colonies retained a radial-type morphology (Figure 3C). Interestingly, when they were subcultured, all the spherical-type colonies (clone #4 and #8) either differentiated or died, whereas all the radial-type colonies proliferated except for clones #2 and #3 (these two cell lines died during maintenance). 

### 3.4. Characterization of Pluripotency Markers in Primary ciPSC Colonies

**Figure 4:**
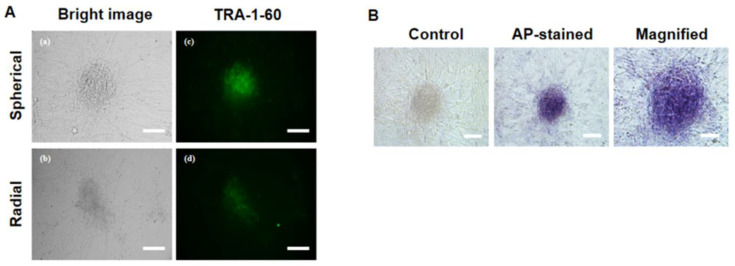
Immunocytochemistry of pluripotency markers in primary canine induced pluripotent stem cell (ciPSC) colonies. (**A**) Expression of the pluripotency marker TRA-1-60 in ciPSCs, as assessed by immunocytochemical analysis. (a) and (c) A spherical colony (No. #4 colony). (b) and (d) A radial colony (No. #2 colony). Scale bars = 50 μm. (**B**) Image of a spherical domed ciPSC colony (No. #4 colony) after introducing four factors (*VEE-hOct4*, *VEE-hKlf4*, *VEE-hSox2*, and *VEE-hGlis1*) by mRNA transfection on day 27 (left); scale bars = 200 μm. Representative image of an AP-positive colony (middle); scale bars = 200 μm. Magnified image of an AP-positive colony (right); scale bars = 50 μm.

To evaluate the quality of the putative primary ciPSC colonies, the expression of the pluripotent stem cell marker TRA-1-60 was assessed by live-cell imaging. Both the spherical- and radial-type colonies expressed the TRA-1-60 pluripotency marker, as shown in (Figure 4A). AP activity was also observed in a spherical colony (Figure 4B).

**Figure 5:**
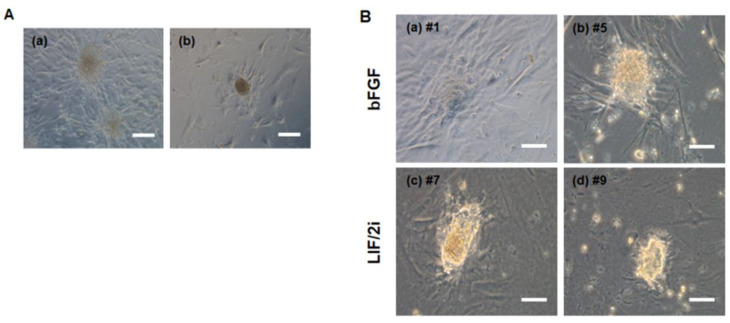
Morphological changes of canine induced pluripotent stem cell (ciPSC). (**A**) Representative images of radial colonies. (a) Radial colonies of putative ciPSCs were appeared at day 26 after VEE RNA transfection. (b) At passage 1, a flat and rounded colony appeared. Scale bars = 100 μm. (**B**) Morphological changes of ciPSCs culture in the presence of basic fibroblast growth factor (bFGF) or leukemia inhibitory factor (LIF)/ PD0325901 and CHIR99021 (two inhibitor; 2i). (a, b) The attached clumps appeared under bFGF-only conditions. (c, d) The culture conditions were changed to LIF/2i. Scale bars = 200 μm.

The radial colonies were subcultured and maintained well on the feeder layers. When the radial colonies were subcultured, almost of them have changed their morphology from a flat shape to a round shape (Figure 5A), and several colonies spontaneously differentiated in the presence of only 10 ng/mL of bFGF.

To determine the optimal culture conditions, the 10 ng/mL of bFGF culture condition was changed to 1000 units of mLIF + 0.5 μM MEK1/2 inhibitor (PD0325901) + 3 μM of GSK3β inhibitor (CHIR99021) (LIF/2i). Both culture conditions were compared to investigate which condition was more appropriate. Six days later, the flat or round-shaped colonies had changed in appearance to dome-shaped colonies and gradually proliferated under the LIF/2i condition (Figure 5B). However, these results should be further examined to determine whether such ciPSCs are LIF-dependent. These data indicated that the primary ciPSC lines generated using RNA-based reprograming were maintained well under the LIF/2i condition.

### 3.5. Gene Expression Analysis of Putative ciPSCs by RT-PCR

**Figure 6:**
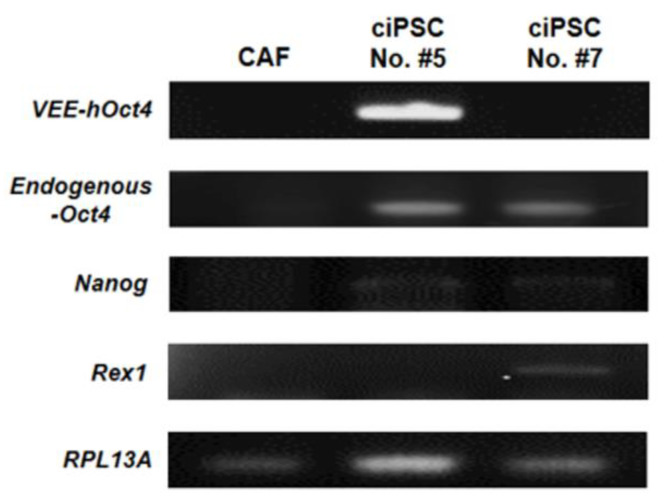
Gene expression analysis of the putative canine induced pluripotent stem cell (ciPSC) lines. The expression of *RPL13A*, *VEE-hOct4*, endogenous *Oct4*, *Nanog,* and *Rex1* mRNAs was analyzed in the putative ciPSC colonies on day 17 by RT-PCR. The *RPL13A* gene served as a reference gene; *VEE-hOct4* served as an exogenous gene; *Oct4, Nanog*, and *Rex1* served as pluripotency marker genes.

The total eight radial colonies were cultured to passage 3, so we investigated the gene expression levels by RT-PCR. The putative ciPSCs in the transition or intermediate stage did not grow well after passage 3, so it was difficult to obtain a sufficient cDNA concentration to perform a gene expression analysis. Of the total eight cell lines maintained until passage 3, we analyzed the level of gene expression by PCR using the ciPSC #5 cell line and the ciPSC #7 cell line, with a relatively sufficient cDNA concentration. To characterize putative ciPSCs, the expression levels of *RPL13A* (a reference gene), *VEE-hOct4*, *VEE-hKlf4*, *VEE-hSox2*, *VEE-hGlis1*, *Oct4*, *Nanog*, and *Rex1* mRNA were measured by RT-PCR (Figure 6 and Table S1). Among the exogenous *VEE-hOct4*, *VEE-hKlf4*, *VEE-hSox2*, and *VEE-hGlis1* transcripts, only *VEE-hOct4* was expressed in the putative ciPSC #5 cell line. The expression of the endogenous *Klf4*, *Sox2*, and *Glis1* genes was not detected (data not shown), but the endogenous *Oct4* and *Nanog* genes were expressed in both cell lines. The putative ciPSC #7 cell line also showed the expression of *Rex1*, which is a well-known ground-state (naïve) pluripotent marker gene [43]. The results of this study suggest that the ciPSC lines generated using RNA-based reprograming were in the late intermediate stage of reprogramming.

## 4. Discussion

**Figure 7:**
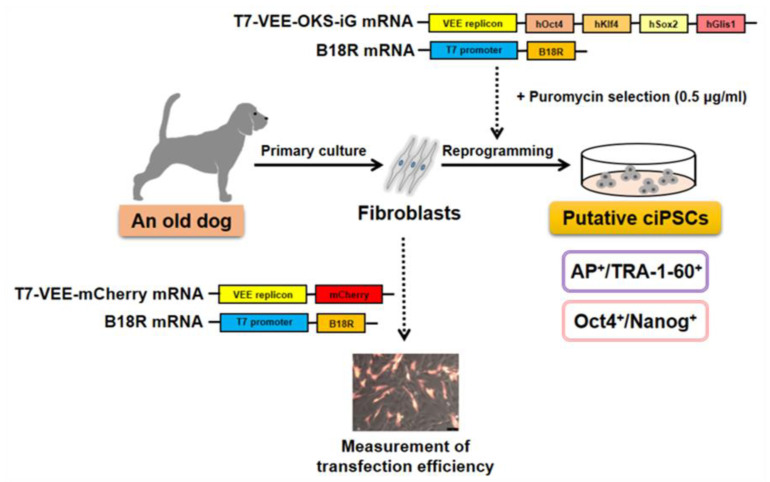
Schematic diagram showing the generation of putative induced pluripotent stem cells (iPSCs) from canine adult fibroblasts (CAFs) using synthetic RNAs. In this study, the fibroblasts obtained from an old dog were reprogrammed using a non-integrating VEE RNA virus replicon which has four reprogramming factors (collectively referred to as *T7-VEE-OKS-iG*) and co-transfected with the *B18R* mRNA. To evaluate the transfection efficiency, CAFs were co-transfected with *T7-VEE-mCherry* mRNA and *B18R* mRNA on day 1, using the RiboJuice™ transfection reagent. One day after the final transfection, the cells were selected with puromycin (0.5 µg/mL) until day 10. After about 25 days, the putative canine iPSC colonies were identified showing TRA-1-60 expression and alkaline phosphatase (AP) activity. Finally, the expressions of endogenous pluripotency markers such as the *Oct4* and *Nanog* transcripts were confirmed, suggesting that the generated canine iPSCs were in the late intermediate stage of reprogramming.

The generation of iPSCs using an integration-free VEE RNA replicon system has great potential for clinical research [26,27,44] due to concerns over the integration of DNA vectors into the genome. To generate integration-free iPSCs from the fibroblasts of a 13-year-old dog, we used an efficient VEE RNA-based iPSC generation strategy. Our findings suggest a new method to utilize the VEE RNA approach for canine somatic cell reprogramming regarding the transfection and reprogramming efficiency (Figure 7).

The VEE RNA replicon used in the experiments contains the puromycin resistance gene. Thus, the transfected cell lines have been isolated by puromycin selection. The typical puromycin working concentration range is 0.5 to 10 μg/mL for mammalian cells [45], and the optimal concentration should be determined empirically before selecting transfected cells. Hong et. al. have reported that stably transfected canine fetal fibroblasts were selected with 2 μg/mL of puromycin for 2 weeks [46]. Techangamsuwan et al., on the other hand, have transfected canine schwann cells and olfactory ensheathing cells, and the stable transfectants were selected with 0.4 μg/mL of puromycin [47]. Therefore, the puromycin sensitivity varies according to the target cell type, so an appropriate concentration should be determined prior to antibiotic selection. In this study, the optimal puromycin concentration for the selection of CAFs transfected with VEE-OKS-iG synthetic mRNA was found to be 0.5 μg/mL. In addition, a previous study showed that the activation of innate immune responses enhanced the efficiency of iPSC generation by repeated mRNA transfection [48]. It has also been shown that VEE RNA induces especially strong interferon (IFN)-α/β innate immune responses [26]. To reduce the innate immune response to VEE RNA, the B18R protein was used, which binds to and neutralizes type 1 IFNs [40]. Interestingly, the cells co-transfected with *T7-VEE-mCherry* RNA and *B18R* RNA expressed mCherry at higher levels than the cells transfected with *T7-VEE-mCherry* alone. Therefore, the B18R protein mitigated VEE RNA-induced immune responses and increased the transfection efficiency.

According to previous studies, the pluripotent states were classified into two states, referred to as the “naïve” and “primed” states [33,49,50]. The naïve PSCs typically retain small, compact, dome-shaped colonies, whereas the primed PSCs display a large and flattened monolayer morphology. In this study, 12 colonies were derived from CAFs following only one transfection experiment using the VEE RNA system. On the basis of their morphology, these initial colonies were classified into two types, spherical and radial. Neither are a typical morphology of ciPSC colonies, composed of tightly packed dome-shaped cells surrounded by a distinct border similar to canine ESCs. Several researchers have identified those colonies, showing unclear margins and radial colony morphologies as a partially reprogrammed cell [51,52,53]. Even though both the spherical and radial colonies stained positive for the pluripotency marker TRA-1-60 by immunocytochemistry, they might be partially reprogrammed [10,54,55]. In addition, David et. al. have reported that AP is detected during the early intermediate stage of reprogramming [56]. Because our results have also shown AP activity in initial colonies through AP staining, these cell lines can be considered to have passed the early intermediate stage of reprogramming. Especially, all the spherical colonies (No. #4 and No. #8) differentiated or died when they were subcultured, whereas all the radial colonies proliferated after subculture except for clones #2 and #3, suggesting that the radial-type colonies were maintained better than the spherical-type colonies.

Naïve and primed PSCs differ substantially in their major pluripotency-related signaling pathways: the LIF-dependent naïve state and the bFGF-dependent primed state, both of which are commonly reported in mouse and human cells [57]. Even though both signaling pathways seemed to be mutually exclusive, several reports have shown that most ciPSCs require both bFGF and LIF supplementation for proliferation in the undifferentiated state [32,36,37,54], whereas only one report has shown that ciPSCs were dependent on LIF alone [10]. The LIF-dependent ciPSCs seemed to be established in the naïve state. Whitworth et al. [10] also reported that ciPSCs could be cultured well in a cocktail medium supplemented with a cocktail of small molecules containing not only bFGF and LIF, but also GSK3β inhibitor, MEK1/2 inhibitor, TGF-β antagonist, and valproic acid. Therefore, we also compare the numerous different culture conditions for RNA-based reprogramed ciPSCs established in this study. Although additional study should be performed to determine whether these ciPSCs are LIF-dependent or not, the evaluation of different culture media for the ciPSC lines revealed that those based on the LIF/2i condition are more suitable for both the isolation and expansion of ciPSCs compared to the bFGF culture condition.

Recently, RNA-based iPSC approaches using four individual reprogramming-factor mRNAs generated by IVT were noted [41,58,59]. However, due to the rapid degradation of reprogramming factor mRNAs, this RNA-based reprogramming method required daily transfections during the reprogramming period or that the mRNA transfection be continued for a few days to increase the transfection efficiency [60]. Therefore, the transfection efficiency was determined as a function of the transfection frequency in this study. As the frequency of transfection increased, primary colonies appeared to be reprogrammed more efficiently because they were observed at a much higher percentage, whereas the cells transfected only once were speculated to have reached the transition state (data not shown). Since there is a self-replicative VEE replicon in the vector used in the experiment, only one transfection was attempted without continuous transfection. Using this system, the reprogramming factor mRNAs replication and transcription may continue to occur in transfected cells once [61]. Even though mRNA delivery to CAFs has been conducted several times, the initial colonies were speculated to have been partially reprogrammed and were potentially in the transition state. The main cause of entry into the transition state by these primary colonies is as follows. Despite the potential of efficient mRNA transfection for reprogramming, cellular senescence served as a fundamental barrier against fully reprogramming fibroblasts from aged dogs to iPSCs [62,63]. Moreover, a more efficient transgene-delivery system was required for successful reprogramming. Previous data has shown that electroporation was more suitable for producing stable cell lines than lipofection [64].

Recent reports have shown the cellular changes that occur during the reprogramming process of MEFs [33,65,66]. The reprogramming process can be classified into two phases—namely, an early “stochastic” and a following “deterministic” phase [65]. If the reprogrammable cells enter an intermediate state (i.e., a transition state), then the cells go through a stochastic activation of pluripotency genes [67], the transient activation of developmental regulators [68], and the activation of glycolysis [69,70]. One thing to be sure is that an intermediate phase of morphological changes from somatic cells to iPSCs during reprogramming must occur, during which reprogrammable cells undergo transcriptional and epigenetic genetic changes [61,71]. The stably expressed reference genes differ with each cell line, and care should be taken when selecting an appropriate reference gene for RT-PCR. Although glyceraldehyde-3-phosphate dehydrogenase (*GAPDH*) is the most commonly used reference gene, many reports have demonstrated that this gene is not stably expressed in different tissues [72,73]. Instead, it was reported that *RPL13A* is more stably expressed as a reference gene in whole canine skin than *GAPDH* [72]. Using *RPL13A* as a reference gene, the expression levels of exogenous (*VEE-hOct4*, *VEE-hKlf4*, *VEE-hSox2*, and *VEE-hGlis1*) and endogenous (*Oct4, Nanog*, and *Rex1*) genes were determined in the putative ciPSC colonies and CAFs. In this study, endogenous *Oct4*, *Klf4*, *Sox2*, and *Glis1* genes were analyzed in the putative ciPSCs by RT-PCR, but those endogenous genes were not fully activated in these cell lines except for the *Oct4* gene. Generally, many researchers have reported that the expression of the endogenous expression of *Oct4* might be detected from the early transitional stage of reprogramming [65,71,74,75], whereas the endogenous expression of *Sox2* could be detected only after transition stage, particularly in the stabilization stage of reprogramming [56,71,76]. However, little is known about the transitional state of canine reprogramming process. The expressions of the *Oct4* and TRA-1-60 with AP activity without the endogenous expression of the *Sox2* in the ciPSCs generated by the VEE-RNA system suggest that they are in a late intermediate or transition state, and not in the stabilization stage of reprogramming. To our knowledge, we are the first group demonstrating a significant improvement in ciPSCs in a transitional state.

In previous studies, the expression of endogenous factors such as *Oct4*, *Klf4*, *Sox2*, and *c-Myc* was analyzed in ciPSCs [7,31], but these primer sequences are not suitable for exogenous and endogenous gene expression analysis because they overlap with those of humans. Unlike mouse and human cells, the development of canine-specific markers is insufficient and some gene sequences are very similar between dogs and humans, making it difficult to perform gene-expression analysis because only a few certain canine gene sequences are found in the GenBank^®^ (database; http://www.ncbi.nlm.nih.gov). In addition, only the exogenous *VEE-hOct4* gene was expressed in the putative ciPSC #5 cell line, suggesting that this cell line was more partially reprogrammed than the No. #7 cell line because the overexpressed exogenous Oct4 genes remain. Although the endogenous *Oct4* (strong expression) and *Nanog* (weak expression) genes were expressed in both cell lines, *Rex1*, known to be important for the naïve pluripotent state [43,77], was clearly observed only in the No. #7 cell line. Although all of the colonies generated in this study showed a strong expression of *Oct4*, almost of the colonies (eight cell lines including #5) showed the weak expression of *Rex1*, except for No. #7 ciPSCs. The weak expression level of *Rex1* with the strong expression of “*Oct4*” in these colonies suggests that those colonies are not in the ground state of pluripotency [49], unlike the #7 ciPSCs, which show the most “naïve” pluripotency. These results indicate that the almost of the ciPSC colonies generated using RNA-based reprograming were in the transition stage of reprogramming. The lack of silencing of exogenous transgenes has been reported as a well-known feature of incomplete reprogramming [78]. This indicates that VEE RNA was not fully transfected into CAFs because the size of VEE replicon is very large (11 kb), and a strong immune response was induced by the modified mRNA during reprogramming. Taken together, these findings suggest that the putative ciPS cell lines may be in a late intermediate stage of reprogramming, and in particular the No. #7 cell line is closer to the ground state of pluripotency than the No. #5 line. A limitation of our findings is that the ciPSCs generated in this study subcultured well up to five serial passages while keeping their incomplete pluripotency. Several researchers have previously described that a self-replicating VEE RNA vector is another transgene delivery method for viral-free iPSCs [26,44,79]. However, further studies are required to set up the complete reprogramming and optimize the culture conditions [80] for more long-term culture. It is also necessary to further study the mechanism from the transition state to the fully pluripotent state. This is a preliminary study and requires repeating with quantitative methodologies.

## 5. Conclusions

This work describes a new method for transfecting dog somatic cells. To our knowledge, we are the first group to report synthetic RNA-based reprogramming for ciPSCs from the fibroblasts of an old dog. We performed reprogramming with the somatic cells of a Korean native dog, an old Jindo dog, and it was possible to determine the optimal concentration of puromycin treatment suitable for the selection of transfected individual cells and to present the results of an RNA transfection efficiency measurement. It is speculated that the reason why the established putative ciPSC lines remained only in the early passage was that they were partially reprogrammed or at an intermediate reprogramming stage, but further experimentation is needed to prove this. Although further studies are needed to characterize the conversion of canine somatic cells to ciPSCs using synthetic RNAs, these results may be useful for developing canine-specific markers to characterize iPSCs in the transition or deterministic phase. Furthermore, the reprogramming method using non-integrating, self-replicative VEE RNA will help us to generate clinically applicable ciPSCs.

## Supplementary Materials

The following are available online at https://www.mdpi.com/2076-2615/10/10/1848/s1: Table S1: Primers list for RT PCR.
