# Supplementary material for: Optimized Approaches for the Induction of Putative Canine Induced Pluripotent Stem Cells from Old Fibroblasts Using Synthetic RNAs
Source: Animals (Basel). 2020 Oct 11;10(10):1848. doi: 10.3390/ani10101848 (PMC7601034; doi:10.3390/ani10101848)
Supplement: Supplementary file 1 [file animals-10-01848-s001.pdf]

Supplementary Table S1. Primers list for RT PCR.

mRNA	Primer sequences	Product size (bp)	GenBank accession number
RPL13A	F: 5'-AAGCAAGAAAGACCTGGAAA-3'<br>R : 5'-ACAGGGATAGCACAAGGGTA-3'	206	NM_001313766
VEE-hOct4	F : 5'-CACCTGGCTTCGGATTTCG-3'<br>R : 5'-AATGCATGGGAGAGCCCAGA-3'	1066	NM_002701
VEE-hKlf4	F : 5'-TTTCTCCACGTTCGCGTCTG-3'<br>R : 5'-GGTCCGACCTGGAAAATGCT-3'	1352	NM_001314052
VEE-hSox2	F : 5'-TGATGGAGACGGAGCTGAAG-3'<br>R : 5'-GGTTCTCCTGGGCCATCTT-3'	180	NM_003106
VEE-hGlis1	F : 5'-GAGAAGCCCAACAAGTGCAT-3'<br>R : 5'-TGCTGGTTCAGCCATCCG-3'	837	XM_017000410
Rex1	F : 5'-AGCATGGACTTATCCAATCC-3'<br>R : 5'-CTCCCACATTCTGCACATAC-3'	197	XM_003639567
Endogenous Oct4	F : 5'-TGGCTGAGCTTCCCGGG-3'<br>R : 5'-CTCGTTGCGAATAGTCACTGC-3'	790	XM_538830
Nanog	F : 5'-GCTGCTATGGACAACATGAT-3'<br>R : 5'-ACCCTTCACCGTTAATTGAG-3'	189	XM_022411387

F: Forward, R: Reverse
